# Clinical, endocrinological, and molecular genetic characterization of Kallmann syndrome and normosmic idiopathic hypogonadotropic hypogonadism in childhood and adolescence

**DOI:** 10.1186/1687-9856-2015-S1-P114

**Published:** 2015-04-28

**Authors:** Sun-Jeong Shin, Ja Hye Kim, Ja Hyang Cho, Jae Hyun Kim, Gu-Hwan Kim, Jin-Ho Choi, Han-Wook Yoo

**Affiliations:** 1 Department of Pediatrics, Asan Medical Center Children’s Hospital, University of Ulsan College of Medicine, Seoul, Korea; 2 Medical Genetics Center, Asan Medical Center Children’s Hospital, University of Ulsan College of Medicine, Seoul, Korea; 3 Department of Pediatrics, Inje University, Ilsan Paik Hospital, Seoul, Korea

## Aims

Isolated gonadotropin-releasing hormone (GnRH) deficiency (IGD) is characterized by delayed or absent sexual development associated with low gonadotropin and sex steroid levels. IGD is classified as Kallmann syndrome (KS) with anosmia and normosmic idiopathic hypogonadotropic hypogonadism (nIHH). This study was undertaken to investigate clinical and endocrinological profiles in patients with KS and nIHH during childhood and adolescence in Korea.

## Methods

Twenty nine patients (24 males and 5 females) were included. Their clinical, endocrinological, and radiologic findings were analyzed by retrospective medical record review. In all patients, all exons and intronic flanking regions of the GNRH1, GNRHR, KISS1, KISS1R, PROK2, PROKR2, TAC3, TACR3, FGF8, FGFR1, KAL1, CHD7, and SOX10 genes were amplified by PCR with specific primers and directly sequenced.

## Results

Of 29 patients, 14 were KS and 15 were nIHH. At diagnosis, mean chronologic age was 16.55 ± 3.76 years; height SDS was -0.92 ± 1.53; testis volume was 2.10 ± 1.25 mL; and Tanner stage was 1.45. There were associated anomalies in some KS patients: 6 patients with hearing loss, 4 patients with congenital heart disease, and 2 patients with cryptorchidism. Absence or hypoplasia of the olfactory bulb or sulci was found in 9 (82.82%) KS patients. Peak LH and FSH levels were 5.64 ± 5.83 and 5.11 ± 4.61 mIU/mL after GnRH stimulation. Baseline testosterone levels were 0.46 ± 0.62 ng/mL. Twenty four patients (20 males, 4 females) received hormone replacement therapy for 51.1 months of duration. During the follow-up period, all patients reached final adult height of 173.29 ± 8.07 cm (0.27 ± 1.03 SDS). Of 29 patients from 28 unrelated families, molecular defects were identified in 6 patients from 5 families (17.9%, 5/28 pedigrees) in KAL1, SOX10, and CHD7. Two male siblings with KS harbored a novel heterozygous mutation of p.W380* in KAL1. Two unrelated female patients with typical and partial/incomplete CHARGE syndrome were heterozygous for frameshift (p.A608Gfs*4) and nonsense (p.W983*) mutations in CHD7, respectively. A 31-year-old male with KS and deafness harbored a heterozygous p.M112I mutation in SOX10.

## Conclusion

This study described clinical, endocrinological, and molecular genetic features in relatively large cohort of IGD patients. Although the mutation screening was performed in ten genes responsible for causing IGD, molecular defects were identified in relatively small proportions of cohort.

